# CDKN2AIP-induced cell senescence and apoptosis of testicular seminoma are associated with CARM1 and eIF4β: CDKN2AIP inhibits the progression of seminoma

**DOI:** 10.3724/abbs.2022040

**Published:** 2022-05-18

**Authors:** Yuming Cao, Zhenlie Chen, Zihan Qin, Kaiyu Qian, Tongzu Liu, Yuanzhen Zhang

**Affiliations:** 1 Department of Gynaecology and Obstetrics Zhongnan Hospital of Wuhan University Wuhan 430071 China; 2 Clinical Medicine Research Center for Prenatal Diagnosis and Birth Health Wuhan 430071 China; 3 Department of Biological Repositories Zhongnan Hospital of Wuhan University Wuhan 430071 China; 4 Urology Surgery Zhongnan Hospital of Wuhan University Wuhan 430071 China

**Keywords:** CDKN2AIP, CARM1, eIF4β, seminoma, senescence, apoptosis

## Abstract

Testicular seminoma is a relatively rare tumor which is mostly detected in male population aged from 15 to 35 years old. Although several molecular biomarkers have been identified to be associated with testicular seminoma pathogenesis, the exact mechanism for testicular seminoma progression remains largely unknown. CDKN2A interacting protein (CDKN2AIP) has previously been identified as a tumor suppressor in multiple malignant diseases. In this study, we aimed to further explore its role in testicular seminoma as well as the underlying molecular mechanisms. Retrospective testicular seminoma clinical samples, normal tissues, NTERA-2 cell line, and mouse xenograft models were used in this study. RT-qPCR, western blot analysis, immunofluorescence microscopy, Co-IP and IP-MS experiments were performed to detect the expression of CDKN2AIP and its interaction with CARM1 and eIF4β. SA-β-gal staining assay and H3K9me3 activity experiments were used to subsequently evaluate the cell senescence and apoptosis. Mouse xenograft animal model was used for *in vivo* study. The results showed that CDKN2AIP is highly expressed in normal testis samples, and is significantly suppressed in testicular seminoma clinical samples and cell line model. Up-regulation of CDKN2AIP is significantly associated with the inhibition of testicular seminoma tumor growth and the increase of cell senescence and apoptosis. CDKN2AIP exhibits anti-tumor activity by interacting with CARM1 and eIF4β. CDKN2AIP induces testicular seminoma cell senescence by suppressing CARM1 expression and eIF4β phosphorylation. The CDKN2AIP-CARM1 and CDKN2AIP-eIF4β interactions, which induce tumor cell senescence and apoptosis, may be the potential druggable molecular pathways in testicular seminoma tumor pathogenesis and progression.

## Introduction

The occurrence of testicular germ cell tumor (TGCT) is quite rare and testicular seminoma is a subtype of TGCT, with a disease occurrence rate less than 1 case in 100,000 people. Testicular seminomas are most diagnosed in male population subgroup aged from 15 to 35 years old [1]. Currently, diagnostic strategies of testicular seminomas include ultrasonography, magnetic resonance imaging (MRI) in combination with pathological examination and serum biological markers [2]. As for therapeutic interventions for testicular seminoma patients, current standard of care includes chemo- or radiotherapy with surgical orchiectomy. It has been suggested that several factors are associated with the prognosis for testicular seminoma patients, including the degree of lymphatic invasion, extra testicular invasion, as well as serum marker levels [3].

Up to date, researchers have identified several recurrent chromosomal anomalies associated with testicular seminoma pathogenesis and progression. Among them, chromosome 12p amplification is the most commonly detected variation in multiple testicular seminoma subtypes [4]. Detailed analysis suggested that amplification of multiple genes on 12p including CCND2, KRAS, and TNFRSF1A, *etc*. plays important roles in the progression of testicular seminomas [ 5, 6].

CDKN2A interacting protein (CDKN2AIP), also known as collaborator of ARF (CARF), is a newly discovered marker involved in tumor pathogenesis. CDKN2AIP has been shown to interact with ARF, which activates the key tumor suppressor gene p53 by ARF-dependent or independent pathways, causing inhibition of tumor growth and tumor cell senescence [ 7, 8]. Interestingly, abnormally-high expression of CDKN2AIP unexpectedly exhibits promotive effects on cancer expansion, and CDKN2AIP promotes cell malignant transformation through transcriptional repression of p21 [9]. Moreover, CDKN2AIP overexpression was also found to induce tumor invasion and metastasis through transcriptional activation of Wnt/β-Catenin and subsequent epithelial mesenchymal transition (EMT) enhancement [10].

We speculated that CDKN2AIP might participate in the physiological process of germ cell proliferation and malignant transformation. Therefore, in this study, we aimed to further investigate the role of CDKN2AIP in testicular seminoma pathogenesis and disease progression.

## Materials and Methods

### Patient recruitment and sample collection

Seminoma samples were provided by Human Genetics Resource Preservation Center of Hubei Province (Department of Biological Repositories, Zhongnan Hospital of Wuhan University, Wuhan, China), a member of International Society for Biological and Environmental Repositories (ID:49623232). Informed consents were signed by all patients. This study followed the ethical rules in accordance with the Declaration of Helsinki and was approved by the Ethical Reviewing Committee of Zhongnan Hospital of Wuhan University (No. 2021009K).

### RNA extraction and qRT-PCR

Total RNA was extracted from cell line samples and clinical samples using RNAiso Plus agent (TaKaRa, Dalian, China) following the standardized protocol. Reverse transcription was performed to generate cDNAs. Reverse transcription product was mixed with qPCR SYBR® Green Master Mix regents (A6001; Promega, Madison, USA) and PCR was run on the ABI 7500 PCR system (Applied Biosystems, Foster City, USA). The mRNA expression levels were calculated using the 2 ^–ΔΔCt^ method and normalized to that of *β-actin*or *GAPDH*. The parameters setting information and primer sequences were listed in Table 1.

**Table:** **Table1** Sequence of primers and T _m_ values used for qRT-PCR

Gene	Primer sequence (5′→3′)	*T*_m_ value (°C)
*CDKN2AIP*	Forward: AGACTAGCACCTCACAGTTGCC	69
Reverse: CACATTGGGACTAAAGCCACC	66
*GAPDH*	Forward: CATCATCCCTGCCTCTACTGG	67
Reverse: GTGGGTGTCGCTGTTGAAGTC	69
*CARM1*	Forward: GGGCTACATGCTCTTCAACG	66
Reverse: GTCCACTCCATGGAAAGATGG	65
*P53*	Forward: CAGCACATGACGGAGGTTGT	68
Reverse: TCATCCAAATACTCCACACGC	65
*Bcl-2*	Forward: GGTGGGGTCATGTGTGTGG	69
Reverse: CGGTTCAGGTACTCAGTCATCC	67
*Bax*	Forward: CCCGAGAGGTCTTTTTCCGAG	68
Reverse: CCAGCCCATGATGGTTCTGAT	67
*β-Actin*	Forward: CATGTACGTTGCTATCCAGGC	66
Reverse: CTCCTTAATGTCACGCACGAT	65

### Cell culture

Testicular seminoma tumor cell line NTERA-2 cl.D1[NT2/D1]CRL-1973™ was acquired from American Type Culture Collection (ATCC, Manassas, USA), cultured in RPMI 1640 medium supplemented with 10% fetal bovine serum (FBS; Hyclone, Logan, USA), 100 IU/mL penicillin and 100 μg/mL streptomycin (Invitrogen, Carlsbad, USA), at 37°C with 5% CO _2_. Human embryonic kidney cell 293 (HEK293) and Human osteosarcoma cell line (U2OS) were acquired from Wuhan HengYiSai biology Co., Ltd (Wuhan, China) and cultured in high glucose DMEM (GIBCO, Grand Island, USA) supplemented with 10% FBS, 100 IU/mL penicillin and 100 μg/mL streptomycin (Invitrogen) at 37°C with 5% CO _2_.

### Western blot analysis

Cells (2×10 ^6^) were washed twice with cold PBS and treated with ice-cold cell lysis buffer RIPA (Beyotime, Shanghai, China) to extract total protein. BCA protein assay kit (P0012S; Beyotime) was used to detect the protein concentration. Protein samples were separated by SDS-PAGE and subsequently transferred onto PVDF membranes (Immobilon-P; Millipore, Billerica, USA). The membranes were blocked with Tris buffer (50 mM Tris, pH 7.5) containing 5% skim milk, followed by incubation with primary antibodies overnight at 4°C. The membranes were rinsed with the Tris-buffered saline and Tween buffer solution (TBST; Sigma-Aldrich, St Louis, USA), and incubated with the corresponding HRP-conjugated secondary antibody for 2 h at room temperature. Protein bands on the membranes were visualized using an enhanced chemiluminescence kit and quantified using ECL chemiluminescence imaging system (Tanon, Shanghai, China). GAPDH was used as the loading control. The primary antibodies used were rabbit anti-CDKN2AIP (1:1000; Abcam, Cambridge, UK), rabbit anti-CARM1 (1:1000; Proteintech, Rosemont, USA), rabbit anti-eIF4β (A5405, 1:1000; Abclonal, Wuhan, China), rabbit anti-p-eIF4β (AP0775, 1:1000; Abclonal), and mouse anti-GAPDH (AC002, 1:8000; Abclonal) antibodies. The secondary antibodies were HRP-goat anti-mouse IgG (1:10,000; Abclonal), HRP-goat anti-rabbit IgG (AS014, 1:10,000; Abclonal).

### Plasmid construction, siRNA synthesis and transfection

*CDKN2AIP* and *CARM1* (coactivator-associated arginine methyltransferase 1) were amplified by PCR using cDNA as template, and inserted into pcDNA3.1 vector (GenePharma, Shanghai, China) for subsequent transfection. The siRNAs used in this study were as follows: si- *Cdkn2aip*, 5′-GCUCAGAGAUCGAGGUGCCCUUGUU-3′, siRNA- *Carm1*, 5′-GCUUUCAUCGGCUCCAUAATT-3′; siRNA-NC, 5′-CAAAGUGACAGAUGCUCCAACCUAU-3′. The siRNAs were designed and synthesized by GenePharma. The cells were seeded in 60-mm dishes and cultured at 37°C and 5% CO _2_ for 24 h. Lipofectamine™ 3000 Transfection Reagent (Thermo Fisher, Waltham, USA) was used for transfection according to the manufacturer’s instructions.

### Co-immunoprecipitation and IP-MS

The co-immunoprecipitation (Co-IP) was performed as described previously [11]. NTERA-2 cells transfected with tagged CDKN2AIP/CARM1 were cultured in 10-cm ^2^ dishes. Cells were lysed in the lysis buffer (20 mM Tris-HCl, pH 7.4, 150 mM NaCl, 1 mM EDTA, pH 8.0, 1% NP-40, and 1× Protease and Phosphatase Inhibitor), then centrifuged at 12,000 *g* for 10 min at 4°C. The supernatants were immunoprecipitated using anti-CDKN2AIP antibody, anti-CARM1 antibody and anti-mouse control IgG (AC011, 1:10,000; Abclonal) at 4°C overnight. The proteins bound by antibody were pulled down by protein A/G magnetic beads which were pre-washed with immunoprecipitation buffer for three times (5 min each). The immunoprecipitates were washed three times with IP buffer and then boiled in 2× SDS loading buffer for 10 min. The boiled samples were subject to SDS-PAGE, followed by mass spectrometry and western blot analysis.

### Immunofluorescence microscopy

Cell slides in 24-well plates were washed twice with phosphate-buffered saline (PBS), and fixed with 4% paraformaldehyde (PFA) for 15 min. Fixed cells were permeabilized with 0.5% NP-40 for 30 min, and blocked with 10% goat serum for 1 h. Subsequently, cells were incubated with primary antibodies including rabbit anti-CDKN2AIP (1:100; Abcam), rabbit anti-CARM1 (1:100; Proteintech), rabbit anti-H3K9me3 (1:100; Cell Signaling, Beverly, USA) at 4°C overnight, and washed three times with PBS (10 min each). Then slides were incubated with goat anti-rabbit IgG H&L-Alexa Fluor® 488 (1:400; Abcam) or goat anti-rabbit IgG H&L-Alexa Fluor® 594 (1:400; Abcam) at room temperature for 1 h, and washed three times with PBS (5 min each). The cell nuclei were stained with DAPI. Finally, slides were examined and images were captured with a confocal microscope.

### Cell apoptosis analysis by flow cytometry

The cell apoptosis rate was analyzed by flow cytometry utilizing Annexin V-fluorescein isothiocyanate (AV-FITC) apoptosis detection kit (BioLegend, San Diego, USA). Briefly, NTERA-2 cells were seeded into 6-well plates (4×10 ^5^ cells/well) and transfected for 24 h in advance. Then the cells were collected and washed with PBS for three times. The cells were harvested and resuspended in 500 μL binding buffer. Annexin V-FITC (5 μL) and PI (5 μL) were added to the buffer, and incubated for 15 min in the dark. Then cells were analyzed by flow cytometry.

### Cell senescence assay

Cell senescence was analyzed using the β-galactosidase staining kit (Beyotime) according to the manufacturer’s instructions. Briefly, after the cells were treated with D-galactose, cells in each group were washed with PBS and fixed with 4% paraformaldehyde for 15 min at room temperature. Then the cells were washed three times with PBS and incubated in SA-β-gal staining solution overnight at 37°C. The senescent cells were observed under light microscope with blue color. At least 300 cells were counted for each group of NTERA-2 cells.

### SCID mice xenograft model

Pathogen-free 8–10 weeks old BALB/c SCID mice were used. For testicular seminoma tumor cell inoculation, cells were harvested by trypsinization and viable cells (5×10 ^6^) were suspended in 1 mL of cell culture medium. An aliquot of 200 μL of cell suspension was injected subcutaneously into each SCID mouse in every treatment group. The mice bearing testicular seminomas were sacrificed when the tumor reached maximal growth (up to 20% of the body weight of the mouse at the beginning of the experiment) or started to ulcerate. Primary tumors were removed, weighed, and fixed in 10% PFA in PBS for subsequent immunohistochemical assay and western blot analysis.

### Statistical analysis

Statistical analysis was conducted using the software package SPSS 21.0 for Windows (IBM-SPSS, Chicago, USA). Data were presented as the mean±SD of three independent experiments. Statistical test of differences between numerical data was performed by standard *t*-test. Pearson test was conducted to compare gene correlation. *P*<0.05 was considered to be statistically significant.

## Results

### CDKN2AIP was specifically detected in human testis while suppressed in testicular seminoma cells

**Figure 1:**
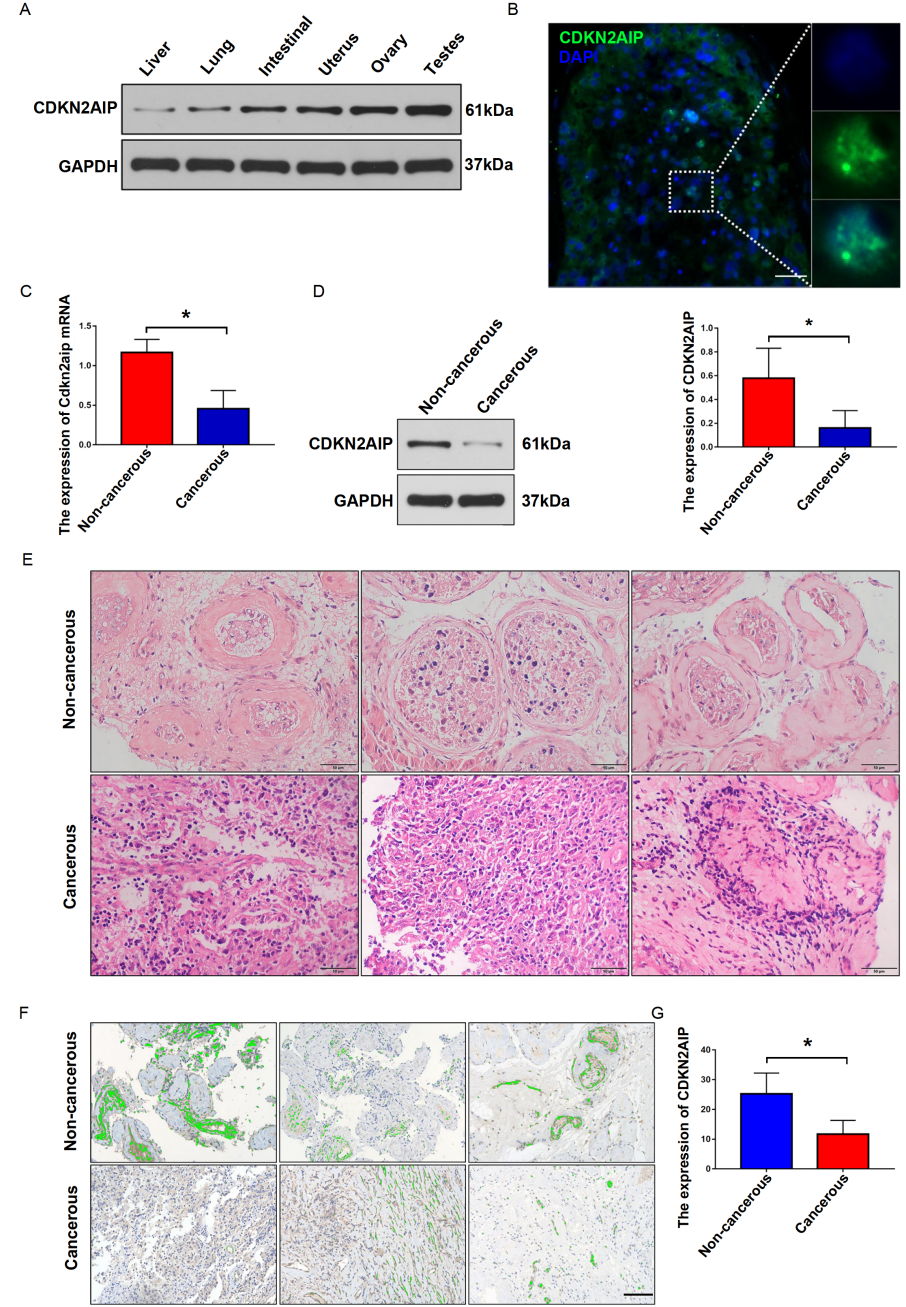
Figure1 High protein expression of CDKN2AIP is involved in the inhibition of seminoma (A) The expression of CDKN2AIP protein in several kinds of human normal tissues, including testes, liver, lung, small intestine, uterus and ovary was detected by western blot analysis. GAPDH serves as the loading control. (B) Immunofluorescence assay showed the localization of CDKN2AIP protein in multiple kinds of cells including spermatogonium, spermatocyte, and sperm in human testis. Scale bar=50 μm. (C,D) The expression of Cdkn2aip at mRNA and protein levels in testicular seminoma tumor and matched adjacent normal tissues was detected by qRT-PCR and western blot analysis respectively. Data are presented as the mean±SD. *P<0.05. (E) HE staining of testicular seminoma tumor tissue and matched adjacent normal tissue. Scale bar=50 μm. (F,G) IHC assay was used to detect CDKN2AIP protein expression in three pairs of matched testicular seminoma tumor and adjacent normal tissues. Data are presented as the mean±SD. *P<0.05. Scale bar=50 μm.

Firstly, in order to understand the special role of CDKN2AIP in human testis, CDKN2AIP protein expression was detected in multiple human organ tissue samples including liver, lung, intestine, uterus, ovary and testis by western blot analysis. Results indicated that the highest expression of CDKN2AIP was detected in human testis sample ( Figure 1A). To elucidate the localization of CDKN2AIP, immunofluorescence assay was performed. The results showed that CDKN2AIP expression was identified in several kinds of cells including spermatogonium, spermatocytes, and sperm cells in human testis tissue ( Figure 1B). Subsequent qRT-PCR and western blot analysis demonstrated that the mRNA and protein level of CDKN2AIP was significantly lower in tumor tissues than in normal tissues ( Figure 1C,D). Moreover, HE staining showed the expression of CDKN2AIP in the testicular seminoma tumor tissue and adjacent non-cancerous tissue ( Figure 1E). Consistently, further IHC staining also indicated that the expression level of CDKN2AIP was significantly lower in testicular seminoma tumor samples than in matched non-cancerous tissue samples ( Figure 1F,G).

### CDKN2AIP interacted with CARM1 to exhibit promotive effects on cell senescence

**Figure 2:**
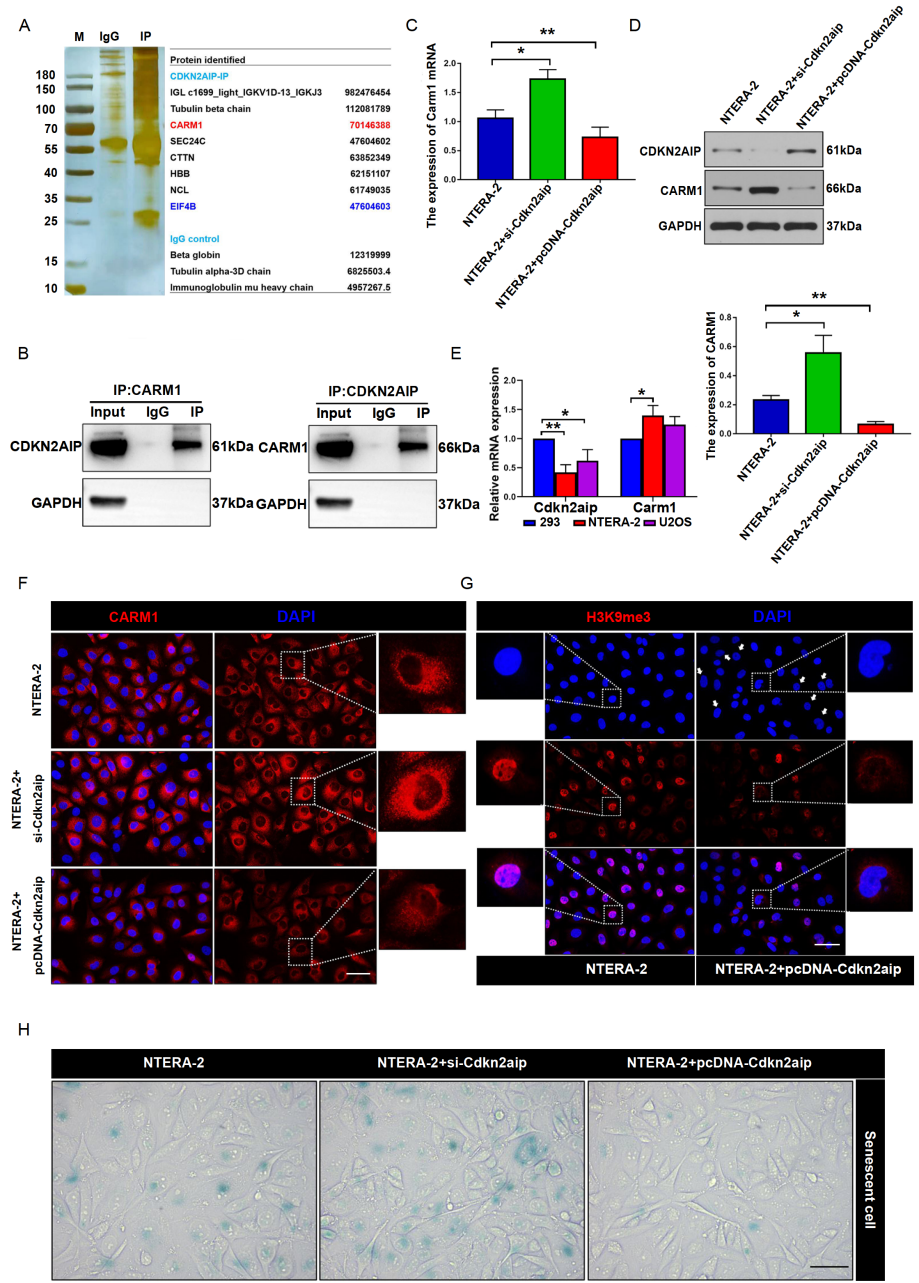
Figure2 CDKN2AIP interaction with CARM1 inhibits the senescence of NTERA-2 cells (A) IP-MS experiment was performed to detect CDKN2AIP-interacting proteins in NTERA-2 tumor cell line. Enrichment fold level of each interacting protein detected in the CDKN2AIP-IP group or IgG control group was listed in detail in the table on the right. (B) Co-IP validation experiment was performed to confirm the interaction between CDKN2AIP and CARM1 in NTERA-2 cell line. GAPDH serves as the loading control. (C,D) qRT-PCR and western blot analysis were used to measure the Carm1 mRNA and protein expression levels in CDKN2AIP siRNA or overexpression NTERA-2 cell groups. Data are presented as the mean±SD. *P<0.05, **P<0.01. (E) mRNA expressions of Carm1 and Cdkn2aip in HEK293 (human normal cells), NTERA-2, and U2OS (human osteosarcoma cell). Data are presented as the mean±SD. *P<0.05, **P<0.01. (F) Immunostaining of CARM1 (red) in Cdkn2aip siRNA transfection or overexpression NTERA-2 cell groups. Scale bar=50 μm. (G) Immunostaining of H3K9me3 (red) in Cdkn2aip siRNA transfection or overexpression NTERA-2 cell groups. Scale bar=50 μm. (H) SA-β-gal activity assay in CDKN2AIP-specific siRNA transfection or overexpression NTERA-2 cell groups. Scale bar=50 μm.

Secondly, to further explore the detailed mechanism of the effect of CDKN2AIP on testicular seminoma pathogenesis, we utilized IP-MS assay in NTERA-2 tumor cell line to detect CDKN2AIP-interacting proteins. Several proteins were identified including CARM1, eIF4β, SEC24C, CTTN, HBB, NCL, and POLR2A ( Figure 2A). Subsequently, the CDKN2AIP-CARM1 protein interaction was confirmed by Co-IP assay ( Figure 2B). Therefore, we further explored the impact of CDKN2AIP-CARM1 interaction on malignant behavior of testicular seminoma tumor cells. As shown in Figure 2C,D, after CDKN2AIP-specific siRNA transfection, the mRNA and protein expression of CARM1 was significantly elevated, while CDKN2AIP overexpression vector transfection notably suppressed CARM1 mRNA and protein expression. Significantly lower expression of CDKN2AIP and higher level of CARM1 were observed in NTERA-2 and U2OS cell lines ( Figure 2E), which are all human cancer cells. Moreover, the consistent results were confirmed on NTERA-2 cell groups by immunofluorescence assay ( Figure 2F). To evaluate the impact of CDKN2AIP expression modulation on tumor cell senescence, histone H3K9me3 level, which reflects the degree of senescence-associated heterochromatin foci (SAHF) formation, was measured by SA-β-gal activity assay and immunofluorescence assay. It was found that the tumor cell senescence was significantly increased in CDKN2AIP-overexpressing NTERA-2 cell group, compared with that in the control cell group ( Figure 2G,H).

**Figure 3:**
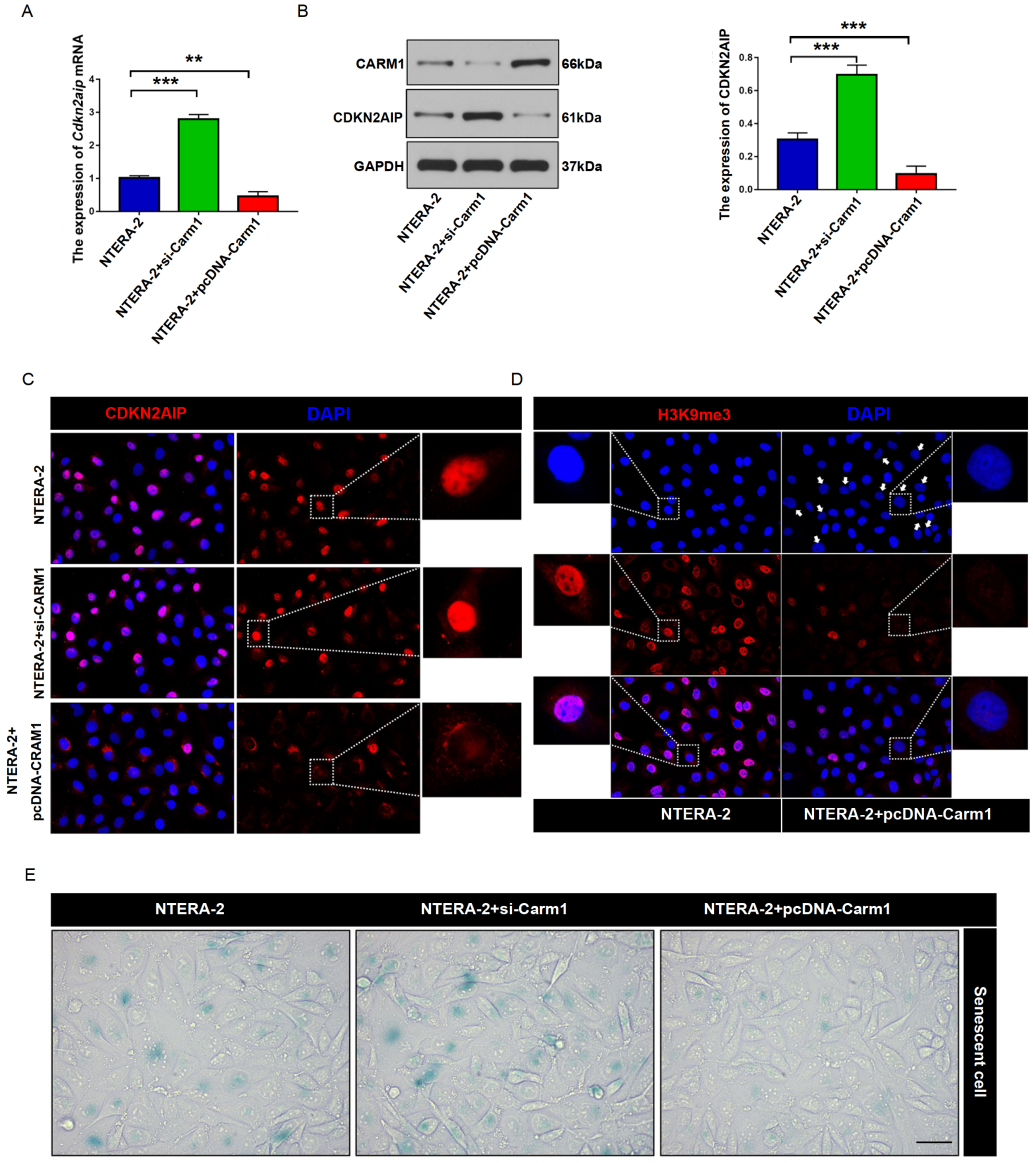
Figure3 CARM1 regulates the expression of CDKN2AIP and NTERA-2 cells senescence (A) qRT-PCR was used to detect the expression of Cdkn2aip mRNA level in Carm1 siRNA transfection or overexpression NTERA-2 cell groups. Data are presented as the mean±SD. **P<0.01, ***P<0.001. (B) Western blot analysis of the expression CDKN2AIP protein in Carm1 siRNA transfection or overexpression NTERA-2 cell groups. GAPDH serves as the loading control. Data are presented as the mean±SD. ***P<0.001. (C) Immunostaining of CDKN2AIP (red) in Carm1 siRNA transfection or overexpression NTERA-2 cell groups. Scale bar=50 μm. (D) Immunostaining of H3K9me3 (red) in Carm1 siRNA transfection or overexpression NTERA-2 cell groups. Scale bar=50 μm. (E) SA-β-gal activity assay on Carm1 siRNA transfection or overexpression NTERA-2 cell groups. Scale bar=50 μm.

Additionally, we further explored the impact of CARM1 modulation on CDKN2AIP expression and its biological significance. The results showed that CARM1 suppression significantly up-regulated CDKN2AIP mRNA and protein expression levels, and vice versa ( Figure 3A,B). Subsequent SA-β-gal activity assay and H3K9me3 immunofluorescence experiment also provided consistent results that CARM1 suppression significantly promoted NTERA-2 cell senescence ( Figure 3C–E).

**Figure 4:**
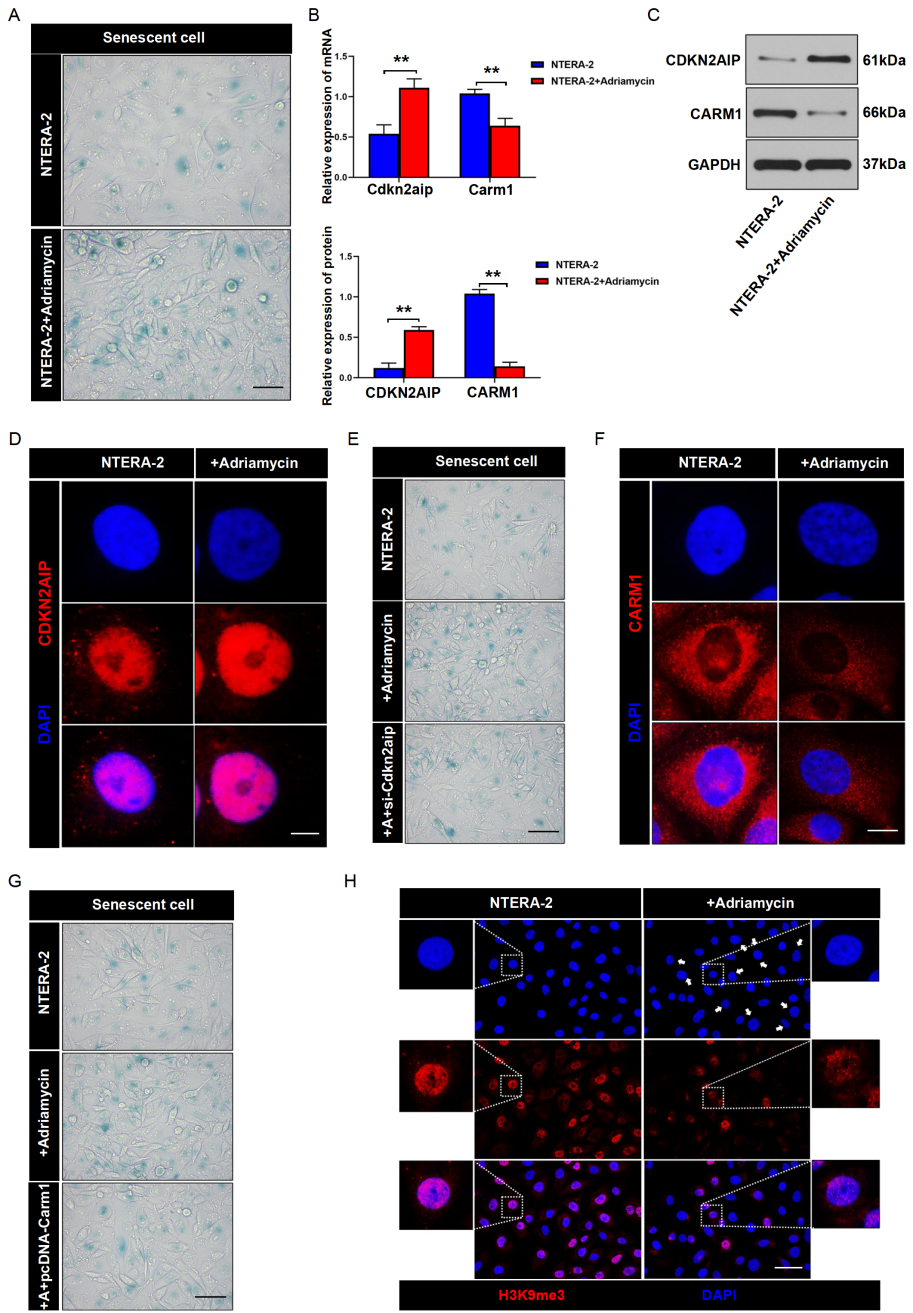
Figure4 CDKN2AIP expression is up-regulated and CARM1 expression is down-regulated in adriamycin-induced senescent cells (A) SA-β-gal activity assay on NTERA-2 cell line. Tumor cells were treated with sublethal concentrations of ADM to induce cell senescence. Scale bar=50 μm. (B,C) qRT-PCR and western blot analysis of CDKN2AIP and CARM1 protein expressions in NTERA-2 cell line. Tumor cells were treated with sublethal concentrations of ADM to induce cell senescence. GAPDH serves as the loading control. Data are presented as the mean±SD. **P<0.01. (D) Immunostaining of CDKN2AIP (red) in NTERA-2 cells. Tumor cell groups were treated with or without sublethal concentrations of ADM to induce cell senescence. Scale bar=50 μm. (E) SA-β-gal activity assay on NTERA-2 cell groups treated with sublethal concentrations of ADM, with or without simultaneous transfection with Cdkn2aip siRNAs. Scale bar=50 μm. (F) Immunostaining of CARM1 (red) in NTERA-2 cells. Tumor cell groups were treated with or without sublethal concentrations of ADM to induce cell senescence. Scale bar=50 μm. (G) SA-β-gal activity assay on NTERA-2 cell groups treated with sublethal concentrations of ADM, with or without simultaneous transfection with CARM1 overexpression plasmid. Scale bar=50 μm. (H) Immunostaining of H3K9me3 in NTERA-2 cells treated with or without sublethal concentrations of ADM to induce cell senescence. Scale bar=50 μm.

To confirm the above findings, a sublethal dose of Adriamycin (ADM) was used to induce cell senescence and the modulative influence of ADM treatment on CDKN2AIP and CARM1 expressions was explored. As shown in Figure 4A,G,H, both SA-β-gal and H3K9me3 immunofluorescence assay indicated that ADM treatment promoted cell senescence. qRT-PCR and western blot analysis results indicated that ADM-treated NTERA-2 cells exhibited an increase of CDKN2AIP and a decrease of CARM1 at both mRNA and protein expression levels ( Figure 4B,C). Immunofluorescence assay also confirmed the enhanced CDKN2AIP expression in ADM-treated NTERA-2 cells ( Figure 4D). Subsequent studies also confirmed that CDKN2AIP-specific siRNA transfection reversed ADM-induced cell senescence ( Figure 4E). Consistently, immunofluorescence assay also confirmed the significantly suppressed CARM1 level in ADM-treated tumor cells ( Figure 4F), and SA-β-gal assay also indicated that ADM-induced cell senescence could be notably reversed by the overexpression of CRRM1 ( Figure 4G).

### CDKN2AIP inhibited anti-apoptotic pathway by binding with eIF4β protein

**Figure 5:**
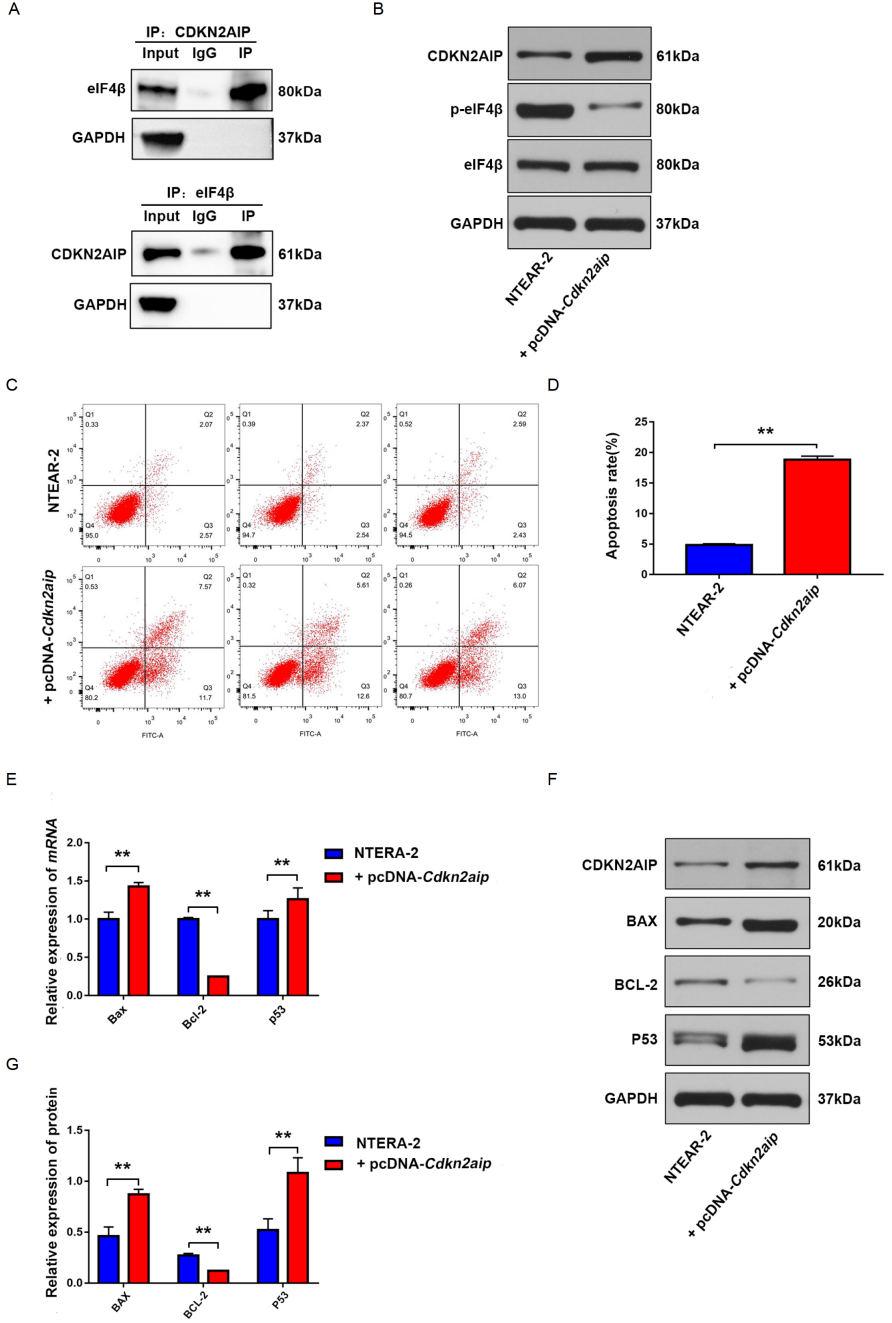
Figure5 CDKN2AIP activates the p-eIF4β/anti-apoptotic proteins pathway by binding to eIF4β (A) The interaction of CDKN2AIP protein with eIF4β was detected by Co-IP assay. GAPDH serves as the loading control. (B) The expression levels of eIF4β and p-eIF4β in CDKN2AIP-overexpressing NTERA-2 cells were detected by western blot analysis. GAPDH serves as the loading control. (C,D) Flow cytometric analysis was performed to evaluate NTERA-2 cell apoptosis, each cell group was transfected with or without CDKN2AIP overexpression plasmids. (D) The statistical analysis of (C). Data are presented as the mean±SD. **P<0.01. CDKN2AIP and p53 plasmids were co-transfected into tumor cells. (E,F) The mRNA and protein expression levels of several cell apoptosis-related markers (Bax, Bcl-2 and p53) in NTERA-2 cells transfected with or without CDKN2AIP specific overexpression plasmids or CDKN2AIP siRNAs. (G) The statistical analysis of (F). Data are presented as the mean±SD. **P<0.01.

IP-MS assay results indicated the interaction of CDKN2AIP with eIF4β, we then performed Co-IP experiment to confirm CDKN2AIP-eIF4β protein interaction ( Figure 5A). Further experiments were designed to investigate the modulative effects of CDKN2AIP on eIF4β expression and the impact of the CDKN2AIP-eIF4β interaction on testicular seminoma tumor pathogenesis and progression. As shown in Figure 5B, western blot analysis indicated that the phosphorylation of eIF4β protein was significantly suppressed in the CDKN2AIP overexpression group compared with that in control NTER-2 cells, while the total eIF4β protein level exhibited no significant change. To further explore the impact of CDKN2AIP-eIF4β interaction on tumor cell apoptosis, flow cytometric analysis was conducted. The results indicated that CDKN2AIP overexpression significantly promoted cell apoptosis ( Figure 5C,D). Subsequent qRT-PCR and western blot analysis experiment also confirmed that CDKN2AIP overexpression significantly elevated the mRNA and protein expression levels of pro-apoptotic gene *Bax* and *p53*, while the expression of anti-apoptotic gene *Bcl-2* was significantly suppressed ( Figure 5E–G).

### CDKN2AIP inhibited tumor expansion in mouse xenograft model

**Figure 6:**
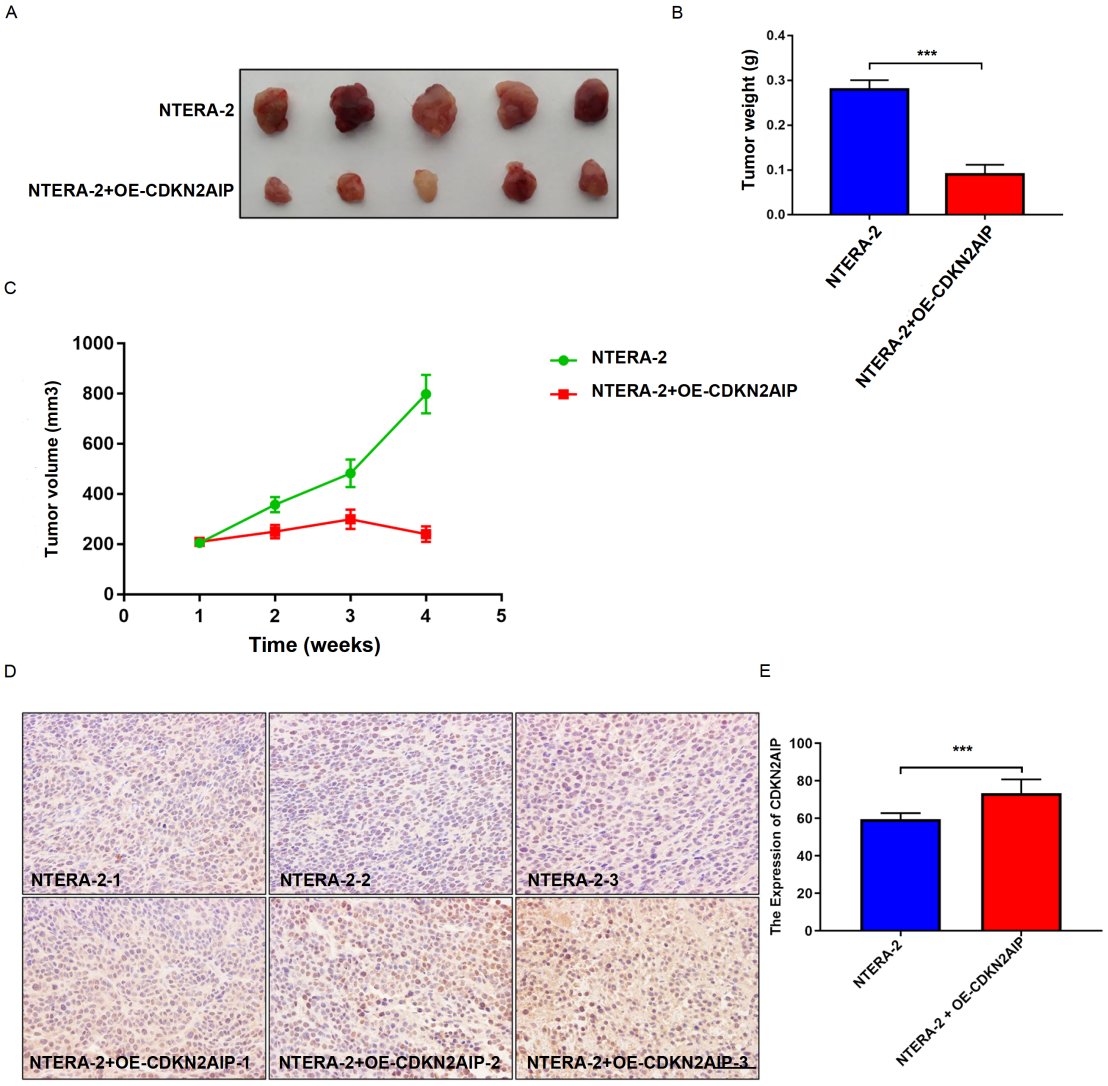
Figure6 Overexpression of CDKN2AIP inhibits tumorigenesis in immune-deficient mice (A) Xenograft model was established by utilizing severe combined immune-deficient (SCID) mice. Each animal group was inoculated with NTERA-2 tumor cells transfected with or without CDKN2AIP overexpression plasmids. (B,C) Tumor volume and weight change in xenograft SCID mice model. Each animal group was inoculated with NTERA-2 tumor cells transfected with or without CDKN2AIP overexpression plasmids. Data are presented as the mean±SD. ***P<0.001. (D,E) Immunohistochemical assay and quantitative analysis of CDKN2AIP expression level in tumor tissue of SCID mice xenograft models. Each animal group was inoculated with NTERA-2 tumor cells transfected with or without CDKN2AIP overexpression plasmids. Data are presented as the mean±SD. ***P<0.001. Scale bar=50 μm.

Finally, in order to confirm our findings *in vivo*, mouse xenograft model was utilized to validate the inhibitory effects of CDKN2AIP on testicular seminoma tumor expansion. Firstly, NTERA-2 cells with or without transfection of CDKN2AIP overexpression plasmids were respectively inoculated into SCID mice, tumor growth was monitored and tumor tissue samples were subsequently collected to detect the CDKN2AIP protein expression level. Tumor expansion results demonstrated that CDKN2AIP overexpression significantly suppressed tumor growth, decreased tumor volume and weight ( Figure 6A–C). Meanwhile, IHC study confirmed that the expression level of CDKN2AIP in xenograft tumor tissue was significantly up-regulated in the CDKN2AIP overexpression group ( Figure 6D,E).

## Discussion

In this study, we demonstrated for the first time that the expression of CDKN2AIP was exclusively high in human testis tissue, and similar expression pattern was also observed in mouse testis tissue. Additionally, sequence homology analysis demonstrated quite conserved CDKN2AIP protein sequences among different species, including human, rat and mouse, which indicated that CDKN2AIP possessed relatively conservative and fundamental role in the regulation of biological function in normal cells. In our study, we also revealed that the suppression of CDKN2AIP was characteristic in testicular seminoma tumor cells, and CDKN2AIP played an inhibitory role in testicular seminoma tumor progression. CDKN2AIP functions as tumor suppressor through two diverse routes: on one hand, CDKN2AIP induces cell senescence by interacting with CARM1, and on the other hand, CDKN2AIP also induces apoptosis by interacting with eIF4β and reduces eIF4β phosphorylation.

Beyond the conventional p53-mediated molecular pathway of CDKN2AIP to induce cell senescence, further detailed study demonstrated that CDKN2AIP exerted its anti-tumor effects through interaction with CARM1 and eIF4β. CARM1, also known as PRMT4, belongs to protein arginine methyltransferase (PRMT) family [12]. CARM1 exerts its regulatory function through methylating residues on multiple transcription factors, RNA polymerase II and other gene expression modulators [13]. Previous studies also indicated that CARM1 is associated with several crucial molecular processes, including RNA processing, transcriptional activation [14], tumor cell apoptosis, cell growth and progression [ 15, 16]. It has been demonstrated that CARM1 upregulation is associated with the pathogenesis of several types of human cancers, including breast cancer, prostate cancer and colorectal cancer, and CARM1 facilitates tumor cell initiation, progression and metastasis [16]. Our study provided evidence that CDKN2AIP exhibits cell senescence-inducing function by suppressing CARM1, which further expands the regulatory molecular network of CDKN2AIP on tumor cell senescence. Meanwhile, CARM1 may also serve as potential therapeutic target for future testicular seminoma treatment. Our findings indicated that knockdown of CDKN2AIP promoted CARM1 expression, and the enhanced expression of CDKN2AIP was found in CARM1-knockdown cells. We speculate that CDKN2AIP may induce CARM1 degradation and a negative regulation between CDKN2AIP and CARM1 may exist. This hypothesis needs to be further investigated.

Moreover, in this study we also demonstrated for the first time that CDKN2AIP exhibited anti-apoptotic functions against tumor cell survival by suppressing the phosphorylation of eIF4β. As an RNA-binding protein, eIF4β stimulates translation through interacting with eIF4A [17], and its activity is modulated by Ser422 or Ser406 phosphorylation through MAPK and PI3K-mTOR pathways [ 18, 19]. However, whether the CDKN2AIP-CARM1 interaction has a cross-talk with eIF4β remains to be explored. Therefore, more detailed studies are required to fully unveil the regulator network of CDKN2AIP in tumor pathogenesis. In addition, due to the nature of CDKN2AIP’s dual functions in both germ cell formation and testicular seminoma pathogenesis, it serves as a representative for a novel cluster of genes named as ‘Cancerous-Testis Gene Cluster’ which may include more candidates.

Notably, as our study was generally based on *in vitro* tumor cell line models and retrospective clinical cohorts, the conclusions drew from our research should be further confirmed in future studies with expanded scale. *In vivo* animal models with CDKN2AIP gene knock-down combined with perspective multi-centered clinical studies are also warranted for future explorations. Adimittedly, only one cell line was used in this study, which is a limitation of this research. Further experiments should be performed using more related cell lines to confirm the conclusions of this study.

In summary, through clinical samples combined with *in vitro* tumor cell line and mouse xenograft model experiments, we demonstrated for the first time that CDKN2AIP induces testicular seminoma cell senescence and suppresses CARM1 expression and eIF4β phosphorylation. The CDKN2AIP-CARM1 and CDKN2AIP-eIF4β interaction-induced tumor cell senescence and apoptosis may be potential druggable molecular pathways in testicular seminoma tumor pathogenesis and progression, which will promote novel therapy development in future testicular seminoma-related research.
